# Integrating scRNA-seq and machine learning identifies MNAT1 as a therapeutic target in OSCC

**DOI:** 10.3389/fimmu.2025.1663487

**Published:** 2025-10-29

**Authors:** Han Gao, Lehua Liu, Weixiang Qian, Yanfei Wu, Jiayao Wang, Weiping Yang, Yinfang Shi

**Affiliations:** Department of Stomatology, First Affiliated Hospital of Huzhou University, The First People’s Hospital of Huzhou, Huzhou, Zhejiang, China

**Keywords:** oral squamous cell carcinoma, machine learning, ubiquitination modification, T cell, MNAT1

## Abstract

**Background:**

Oral squamous cell carcinoma, with high global incidence and mortality, requires improved early intervention strategies. Ubiquitination - a critical post-translational modification - has been strongly implicated in tumorigenesis, with particularly significant roles in T-cell regulation. We developed a T Cell-Related ubiquitination risk model that enhances prognostic prediction and immunotherapy response assessment, offering a framework for personalized OSCC manageme.

**Method:**

T cell-Related Ubiquitination genes were identified based on scRNA-seq analysis, and key genes were selected using WGCNA and LASSO algorithms to construct a prognostic model. Spearman correlation analysis revealed significant associations between riskScore and immune infiltration levels, checkpoint molecule expression, and MMR activity. Pseudotemporal trajectory and cell-cell communication analyses delineated dynamic gene expression patterns driving OSCC progression. Functional validation through colony formation and Transwell assays confirmed the tumor-suppressive effects of key model genes.

**Results:**

Given the high correlation between T cell-Related Ubiquitination genes and the prognosis of OSCC patients, a prognostic model based on patient scRNA-seq data was constructed and validated. The RiskScore derived from our model correlated significantly with expression levels of MMR genes, abundance of immune checkpoint proteins, and immunotherapy response. Cell-cell communication analysis further elucidated epithelial-macrophage crosstalk via MIF and IFN-II signaling, suggesting microenvironment-driven progression mechanisms. *In vitro* functional assays showed that depletion of MNAT1 impaired Cal27 cell proliferation and migration capacity.

**Conclusions:**

Collectively, integrating T cell-Related Ubiquitination genes through advanced computational analyses, we established a robust prognostic model for OSCC and identified MNAT1 as a promoter of malignant progression, highlighting its therapeutic potential.

## 1 Introduction

Oral squamous cell carcinoma (OSCC) represents the most common oral and maxillofacial cancer, with a growing global incidence. Beyond its direct impact on patient mortality and morbidity, the disease incurs significant socioeconomic burdens on affected families and society (1). OSCC pathogenesis arises from a multifactorial interplay, encompassing smoking, alcohol consumption, chronic oral inflammation, HPV infection, oral leukoplakia, and genetic susceptibility (2). Due to the complexity of the pathogenic mechanisms, significant differences in treatment outcomes exist among individuals, underscoring the urgent need for new biomarkers and prognostic models to enable precision medicine (3). In recent years, the rapid development of sequencing technologies has enabled us to delve into individual genomic information, thereby discovering genetic variations and gene expression differences associated with OSCC. These variations and differences not only provide potential biomarkers for disease diagnosis, prognosis prediction, and treatment outcome assessment but also offer robust evidence for guiding the selection of precision treatment strategies (4). The application of single-cell sequencing technology has further expanded the scope of research, revealing not only the interrelation among tumor cells but also providing in-depth insights into the functions of immune cells in the tumor microenvironment, opening up new avenues for the treatment and study of OSCC (5).

T cells, also known as T lymphocytes, are crucial defenders in the immune system, responsible for combating infections, tumors, and autoimmune diseases (6). By identifying and clearing infected cells, finely tuning immune responses, and preserving immune system balance, T cells play a crucial role in the immune system (7). With the rapid progress of single-cell sequencing technology and high-throughput techniques, we have gained a deeper understanding of the subtypes and functions of T cells, which are closely linked to the occurrence and progression of cancer (8). Previous studies have revealed that the interaction between regulatory T cells and neutrophil extracellular traps plays an important role in the carcinogenesis of non-alcoholic fatty liver disease (9). Research by Liang et al. further indicates that tumor-associated Tregs exhibit unique immune features in non-small cell lung cancer, significantly influencing the remodeling of the tumor microenvironment (10). Additionally, upregulation of PD-1 on T cells and its ligand PD-L1 among oral cancer patients is strongly implicated in mediating immune evasion and therapy resistance (11). Consequently, elucidating the interplay between oral cancer and T cells is critical, providing fundamental insights into disease mechanisms, prognostic indicators, and the development of novel therapeutic approaches.

Post-translational modifications (PTMs) of proteins are processes in which specific enzymes or biomolecules chemically modify amino acids after protein synthesis, with diverse forms including phosphorylation, methylation, acetylation and ubiquitination (12). Among them, ubiquitination has attracted considerable attention due to its crucial role in various diseases such as cancer, neurodegenerative diseases, and cardiovascular diseases (13). For instance, ubiquitination mediated by CUL3 and the degradation of BECN1 inhibit the autophagy process, thereby promoting tumor development (14). By abrogating HBx-mediated ubiquitination and degradation of GSK3β, MYH9 knockdown suppresses tumor stemness properties in hepatocellular carcinoma, as reported (15). Furthermore, the suppression of glycolysis and proliferation in OSCC by NEDD4L is mediated through its induction of ENO1 ubiquitination and subsequent proteasomal degradation (16). In this study, we investigates Intersecting genes between ubiquitination-related molecules and T-cell-associated genes. This strategy is anticipated to yield unique insights into ubiquitin-related molecules and its dysregulation in disease states, opening a fertile field for future investigation.

MNAT1 Component of CDK Activating Kinase (MNAT1), as a core subunit of the CDK-activating kinase (CAK) complex, critically regulates cell cycle progression and DNA damage repair (17). Emerging evidence indicates that MNAT1 promotes osteosarcoma pulmonary metastasis via AKT1 upregulation (18). Furthermore, SMYD2-mediated MNAT1 overexpression has been implicated in pancreatic adenocarcinoma tumorigenesis through PI3K/AKT pathway activation (19). It is worth noting that the oncogenic mechanisms of MNAT1 appear to exhibit a certain degree of similarity across different cellular contexts, often involving analogous signaling nodes. Although the role of MNAT1 has been extensively documented in various solid tumors, its expression patterns, functional significance, and underlying mechanisms in OSCC remain largely unexplored. It is still unclear whether MNAT1 operates through a conserved, universally applicable mechanism in OSCC or adopts a unique, tissue-specific oncogenic program. The multifaceted oncogenicity of MNAT1 positions it as a candidate biomarker for prognosis and a promising target for therapeutic intervention in OSCC. Elucidating MNAT1-driven mechanisms in OSCC could provide novel insights into precision oncology strategies.

This study constructs a prognostic model for oral cancer using multiple ubiquitination-related genes and T cell-related genes. Multi-omics analysis combining bulk and single-cell RNA sequencing revealed MNAT1 as a promising prognostic biomarker. Mechanistically, MNAT1 coordinates with tumor-associated macrophages through the MIF and IFN-II signaling axis, synergistically driving OSCC progression via immune microenvironment remodeling.

## 2 Materials and methods

### 2.1 Dataset download

Transcriptional and clinical data related to head and neck squamous cell carcinoma (HNSC) were downloaded from the TCGA database. OSCC samples were then identified based on clinical annotations. Since the clinical annotations of HNSC include detailed anatomical sites, we primarily retained samples originating from the tongue, gingiva, buccal mucosa, lip, floor of mouth, and palate, as confirmed by multiple experienced oral pathologists. These were further intersected with samples having complete clinical information. Our final cohort comprised 336 OSCC samples, including 307 tumor tissues and 29 adjacent normal tissues. Available clinical annotations encompassed survival time, status, age, gender, TNM stage, and other relevant parameters. Furthermore, bulk transcriptome data and scRNA-seq profiles were sourced from the Gene Expression Omnibus (GEO). This included four bulk transcriptomic datasets (GSE41613, GSE30784, GSE74530, GSE31056) and the single-cell RNA-seq dataset GSE172577, which comprises six OSCC specimens.

### 2.2 Single cell sequencing analysis

Single-cell RNA sequencing (scRNA-seq) provides high-resolution transcriptomic profiling at the individual cell level. This technology enables the identification of functional and transcriptional diversity across distinct cell populations and reveals heterogeneity within cell types. Following initial quality control to exclude low-quality samples with nFeature_RNA ≤ 50 or percent_MT ≥ 5%, the filtered data were processed using the “Seurat” package. Principal Component Analysis (PCA) and t-distributed Stochastic Neighbor Embedding (t-SNE) dimensionality reduction facilitated cell clustering. Subsequent cell type annotation was performed with “SingleR”, which allowed us to identify genes specifically associated with T cells.

### 2.3 Cell cycle analysis

Cell cycle analysis was performed using the “Tricycle” R package with human cell cycle reference gene sets. First, a reference-based pseudotime trajectory was constructed from standardized cycling transcriptomes. ScRNA-seq data were then projected onto this trajectory space via the “project_cycle_space” function. Cellular cycle phase positions were quantified by calculating the circular position angle (θ, 0~2π) for each cell using “estimate_cycle_position”. Finally, cell cycle phase distribution across populations was visualized through polar coordinate plots.

### 2.4 Pseudotemporal trajectory analysis

Cellular differentiation trajectories were inferred using the “Monocle2” R package. The computational pipeline initiated with data normalization through the “estimateSizeFactors” function to adjust for intercellular sequencing depth variation, followed by gene dispersion estimation via “estimateDispersions”. Low-abundance transcripts and substandard cellular profiles were systematically filtered to ensure data quality. Inter-subpopulation differential gene expression was subsequently identified using the “differentialGeneTest” function. For trajectory inference, the “DDRTree” algorithm was employed to perform nonlinear dimensionality reduction, projecting high-dimensional transcriptomic data into a low-dimensional manifold. Pseudotemporal ordering of individual cells was ultimately visualized, reconstructing their dynamic progression along inferred developmental trajectories.

### 2.5 Cellchat analysis

Cell-cell interaction networks were systematically interrogated using the “CellChat” computational framework. The analytical pipeline commenced with the construction of a “CellChat” object adhering to the standardized workflow. Leveraging the curated ligand-receptor interaction repository (CellChatDB.human), we subsequently quantified both the interaction probability and network complexity across distinct cellular subsets. This approach enabled the identification of dominant signaling pathways and their topological features within the tumor microenvironment.

### 2.6 WGCNA analysis

weighted gene co-expression network analysis (WGCNA) enables extraction of biologically meaningful patterns from transcriptome-scale expression datasets, elucidating the organization and functional dynamics of gene networks. This advances comprehension of biological processes and offers critical support for disease diagnosis, therapeutic development, and prognostic evaluation. Its applications encompass module discovery, biomarker identification, clinical-module correlation analysis, functional annotation, and network reconstruction. Here, T cell-associated ubiquitination genes served as the foundation for co-expression network construction, with stemness-linked modules prioritized for downstream investigation.

### 2.7 Modeling construction and validation

Applying least absolute shrinkage and selection operator (LASSO) regression, we identified prognosis-associated key genes and established a Cox-based prognostic model. This enabled derivation of individualized riskScores, followed by comprehensive evaluation of these molecular determinants in OSCC patient outcomes.

The riskScore for each OSCC patient is calculated using the following formula: riskScore = Expression of MNAT1 × coefficient + Expression of PSMD10 × coefficient + Expression of EIF3F × coefficient.

We partitioned the TCGA cohort into training and validation dataset, with external validation performed on GSE41613. Subsequent Kaplan-Meier analysis interrogated survival disparities across prognostic groups. Risk-stratified survival curves delineated patient outcomes between high- and low-risk cohorts, while heatmaps visualized differential expression of model genes. To evaluate prognostic predictors, Cox regression modeled associations between riskScore, clinicopathological variables, and survival outcomes. Time-dependent ROC curves quantified predictive accuracy for disease progression. We integrated riskScore with clinical features via nomograms, projecting 1-, 3-, and 5-year survival probabilities. Calibration curves assessed concordance between predicted and observed events, and decision curve analysis (DCA) determined clinical utility of the predictive framework.

### 2.8 Immunoassay

“CIBERSORT” quantified immune cell infiltration abundances across 21 subsets for each OSCC sample, delineating patient-specific immune landscapes. “ESTIMATE” systematically profiled tumor tissue microenvironments through immune, stromal, and ESTIMATE score, comprehensively characterizing tumor mircroenvironment (TME) heterogeneity. To interrogate riskScore-immunotherapy linkages, Spearman correlations assessed associations with mismatch repair (MMR) proteins and immune checkpoints, evaluating predictive potential for therapeutic response. Given microsatellite instability’s (MSI) prognostic and therapeutic relevance, we further compared intergroup MSI scores to stratify immunotherapy beneficiaries. Based on the tumor immune dysfunction and exclusion (TIDE) scoring algorithm, this study used gene expression data from tumor tissues to deeply analyze the immune inhibition and rejection in the tumor immune microenvironment, further verifying the immune therapy response of patients in different risk groups. Additionally, further leveraging the IMvigor210 cohort—with curated transcriptomic profiles and clinical annotations from PD-L1 inhibitor-treated patients—we stratified immunotherapy responses in OSCC sample.

### 2.9 Mutation analysis

Mutation analysis entails detecting, characterizing, and interpreting genomic alterations in biological specimens. These alterations represent heritable changes impacting genomic architecture, protein function, or phenotypic expression. Common variant types include single nucleotide polymorphisms (SNPs), insertions (Ins), and deletions (Del), reflecting distinct DNA modifications. Of particular significance are ATCG substitutions—specific single nucleotide variants (SNVs) that illuminate tumorigenesis mechanisms through genomic alteration signatures. This study will analyze the diverse mutation patterns of patients in high and low-risk groups to explore their underlying mechanisms.

### 2.10 Sensitivity analysis of chemotherapy drugs

Chemotherapy employs cytotoxic agents to combat malignancies through targeted disruption of cancer cell cycle progression. These compounds impair proliferative capacity via diverse mechanisms, ultimately inducing cell death. We systematically profiled eight oral cancer chemotherapeutics—5-Fluorouracil, Paclitaxel, Docetaxel, Entinostat, Cisplatin, Oxaliplatin, Cyclophosphamide, and Carmustine—stratifying differential chemosensitivity between risk groups based on IC50 comparisons.

### 2.11 Functional enrichment analysis

Functional enrichment analysis reveals excessive expression biological pathways and functional modules within gene sets, enabling interpretation of biological significance and regulatory mechanisms. This study covers GO, KEGG, and Hallmark analyses. Specifically, GO analysis was annotated using the c5.go.v7.4.symbols.gmt gene set file. KEGG analysis utilized the c2.cp.kegg.v7.4.symbols.gmt gene set file. Hallmark analysis, on the other hand, was annotated based on the h.all.v2023.2.Hs.symbols.gmt gene set file.

### 2.12 Cell culture

Human oral squamous cell carcinoma-derived CAL27 cells were cultured in DMEM (PM150230, Wuhan Pricella Biotechnology Co., Ltd.) with 10% FBS, 1% penicillin-streptomycin-gentamicin (Beyotime, China), maintained at 37°C and 5% CO_2_.

### 2.13 Cell transfection

CAL27 cells were transfected with Lipofectamine™ 3000 (Thermo Fisher Scientific, USA) per manufacturer’s guidelines. Two short hairpin RNA (shRNA) targeting MNAT1 were designed in **Supplementary Table S1**.

### 2.14 Real-time quantitative PCR

Following established methodology, total RNA was isolated with TRIzol (Invitrogen, USA) (20). After quantification, RNA underwent reverse transcription using EasyQuick RT MasterMix (EasyQuick RT MasterMix, CW2019S, CWBlO, China). Quantitative PCR employed TB Green^®^ Premix Ex Taq™ (Takara Bio, Japan) with GAPDH normalization, applying the 2−ΔΔCt method for expression quantification. Primer sequences are provided in **Supplementary Table S1**.

### 2.15 CCK8 assay

CAL27 cells (2×10^3^/well) were plated in 96-well plates with 100ul complete medium. Cell proliferation was assessed at 0, 24 and 48hours post-seeding by adding 10μl CCK-8 reagent (E-CK-A362, Elabscience Biotechnology Co., Ltd., China) to each well. Post-2h incubation (37°C and 5% CO_2_), 450 nm absorbance was quantified via microplate reader.

### 2.16 Colony formation assays

For colony formation assays, CAL27 cells were seeded in 6-well plates at 1.5×10^3^ cells/well and cultured in DMEM/10% FBS, refreshed every 72 h. Post-10-day incubation, cells underwent PBS washing, 4% paraformaldehyde fixation, and 0.1% crystal violet staining (G1059, Beijing Solarbio Science & Technology Co., Ltd., China). Following three PBS washes, plates were imaged under bright-field microscopy. Colony numbers were quantified using the cell counting module in ImageJ.

### 2.17 Transwell assay

For migration and invasion assays, resuspended in serum-free DMEM, 50,000 CAL27 cells were placed in Transwell upper chambers (NEST Biotechnology Co.,Ltd., China). Lower chambers held 10% FBS-complete medium as chemoattraction source. Post-48h incubation (37 °C and 5% CO_2_), migrated cells underwent 4% paraformaldehyde fixation and 0.1% crystal violet staining. After three PBS washes, non-migrated cells were cleared from membrane surfaces with cotton swabs. Cells were documented by inverted microscopy and quantified via the cell counting module in ImageJ.

### 2.18 Macrophage stimulation and co-culture assay

THP-1 cells (1×106) were treated with 320 nmol/L PMA for 24h. The treated THP-1 cells were then seeded into 6-well plates, with 5×105 CAL27 cells inoculated in the upper chamber. After 48h of culture, macrophages were collected for qRT-PCR.

### 2.19 Data statistics

Bivariate group comparisons used Wilcoxon tests, with Spearman correlations assessing variable associations. Survival outcomes were evaluated by Kaplan-Meier curves and log-rank testing. Cox proportional hazards modeling (“survival” R package) generated hazard ratios (HRs) and 95% confidence intervals (CIs). Statistical significance was defined as two-tailed P < 0.05. All analyses implemented R (v4.2.2).

## 3 Results

### 3.1 Single cell sequencing analysis

**Figure 1:**
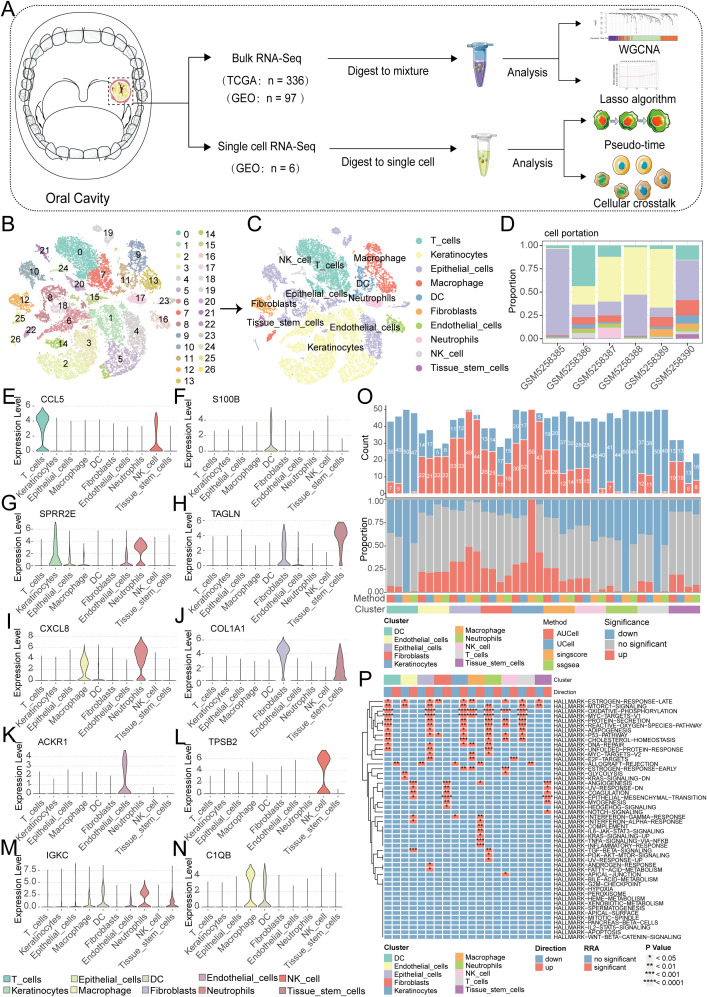
Single-cell sequencing analysis for screening T cell-related genes. **(A)** A flowchart of manuscript. **(B)** PCA and tSNE clustering divided cells into 27 clusters. **(C)** The singleR package annotated cells and categorized them into 10 major cell groups. **(D)** the cell ratios of different samples. **(E-N)** Violin plots illustrate the expression patterns of key genes across distinct cellular subpopulations. **(O, P)** Enrichment analysis via the irGSEA algorithm quantifies dominant signaling pathways within each cell subtype.

**Figure 2:**
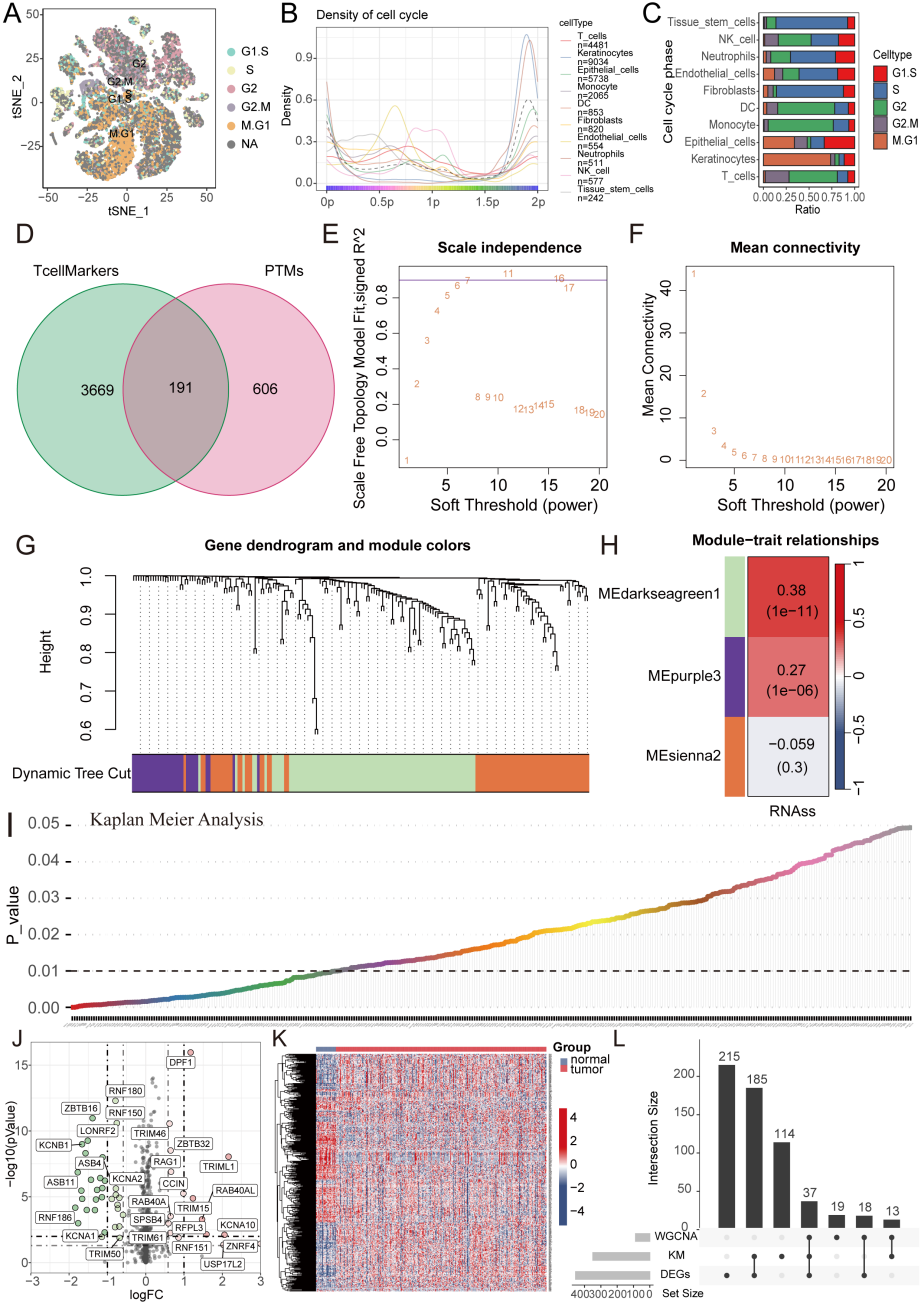
WGCNA combined with differential and prognosis analysis to identify key genes. **(A)** Cell cycle analysis visualizing the phase distribution for each single cell. **(B, C)** Bar plots depicting the proportional representation of distinct cell cycle phases across cellular subpopulations. **(D)** The Venn diagram illustrates the intersection of T cell marker genes and ubiquitin proteasome system genes. **(E, F)** The WGCNA algorithm demonstrates the optimal soft threshold. **(G)** The gene dendrogram displays genes are well clustered into 3 categories. **(H)** MEdarkseagreen1 module genes are found to be closely associated with the tumor stemness index. **(I)** KM analysis shows 349 genes with prognostic value. **(J, K)** The volcano plot and heatmap display 455 differentially expressed genes between cancer tissues and normal tissues. **(L)** The UpSet plot shows the intersection of differential analysis, KM analysis, and WGCNA analysis with 37 genes.

Firstly, a flowchart was created to illustrate the study’s design, implementation, and result analysis processes, enhancing readers’ understanding of the research’s significance (**Figure 1A**). The GEO repository (GSE172577) provided single-cell transcriptomes from OSCC patients, and after data organization, six OSCC samples were included for subsequent analysis. Initially, analysis of nFeature (the number of detected genes) and nCount (the gene expression count per cell), including mitochondrial read fraction per cell, was performed. (**Supplementary Figures S1A–C**). Cells were then filtered based on nFeature_RNA > 50 and percent_MT < 5% for quality control. Additionally, a correlation of 0.89 between nFeature and nCount was observed (**Supplementary Figure S1D**). Following this, genes with significant coefficients of variation between cells were extracted and used for subsequent PCA and tSNE dimensionality reduction analysis, resulting in the classification of samples into 27 clusters (**Figure 1B**, **Supplementary Figure S1E**). These cells were further annotated, revealing distinct categories such as T cells, Keratinocytes, Epithelial cells, Macrophages, DCs, Fibroblasts, Endothelial cells, Neutrophils, NK cells, and Tissue stem cells (**Figure 1C**). Next, we further presented the cell ratios of different samples (**Figure 1D**). Additionally, the main differentially expressed genes among different cell types were displayed (**Figures 1E–N**, **Supplementary Figure S1F**). To delineate pathway heterogeneity among distinct cellular populations, we performed gene set enrichment analysis using the “irGSEA” algorithm. The resultant enrichment profiles revealed cell type-specific activation patterns, with quantitative evaluation of pathway distribution and engagement magnitude across subtypes (**Figures 1O, P**). To interrogate cell cycle deregulation in OSCC, we implemented the “Tricycle” algorithm for cell cycle phase projection. This computational framework accurately mapped single-cell transcriptomes onto a cell cycle phase continuum, enabling systematic classification of proliferative states across the tumor ecosystem (**Figure 2A**). Subsequently, we further demonstrated the different cell cycle ratios of different cell types (**Figures 2B, C**).

### 3.2 Model construction and validation with LASSO-COX algorithm

Given the pivotal role of T cells in oral cancer progression, we extracted 3860 key genes closely associated with T cells, paralleled with a focus on ubiquitination, incorporating 797 key ubiquitination genes. Through the intersection of these two gene sets, we identified a total of 191 key genes (**Figure 2D**). Subsequently, WGCNA analysis was performed, revealing the effective clustering of samples into three modules (**Figures 2E–G**). Tumor stemness index, indicative of tumor cell similarity to stem cells and correlated with enhanced biological activities in stem cells and heightened tumor dedifferentiation, was assessed (21). To probe genes related to this index, we selected the pertinent cluster MEdarkseagreen1 (**Figure 2H**). Further, KM analysis conducted on this gene set unveiled 349 genes significantly associated with prognosis (**Figure 2I**). Following this, differential analysis was executed, culminating in the identification of 455 genes through volcano plots and heatmaps (**Figures 2J, K**). Subsequent gene intersection from WGCNA, differential analysis, and KM analysis yielded a set of 37 genes (**Figure 2L**).

**Figure 3:**
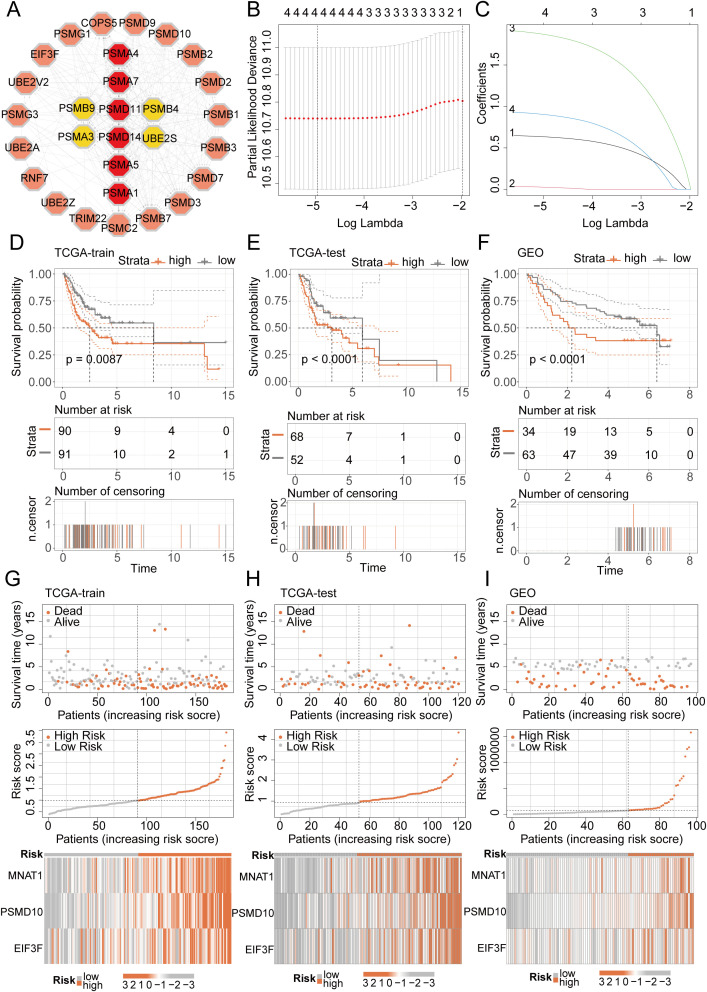
LASSO-COX algorithm constructs a risk prognosis model and validation. **(A)** The PPI network shows the correlation and importance of key genes. **(B, C)** Genes suitable for constructing the optimal model were selected using the LASSO-COX algorithm. **(D-F)** KM analysis revealed that patients in the high-risk group had a worse prognosis than those in the low-risk group in different datasets. **(G-I)** Survival analysis revealed a higher mortality rate in the high-risk group, and the heatmap demonstrated higher expression levels of MNAT1, PSMD10 and EIF3F in the high-risk group.

**Figure 4:**
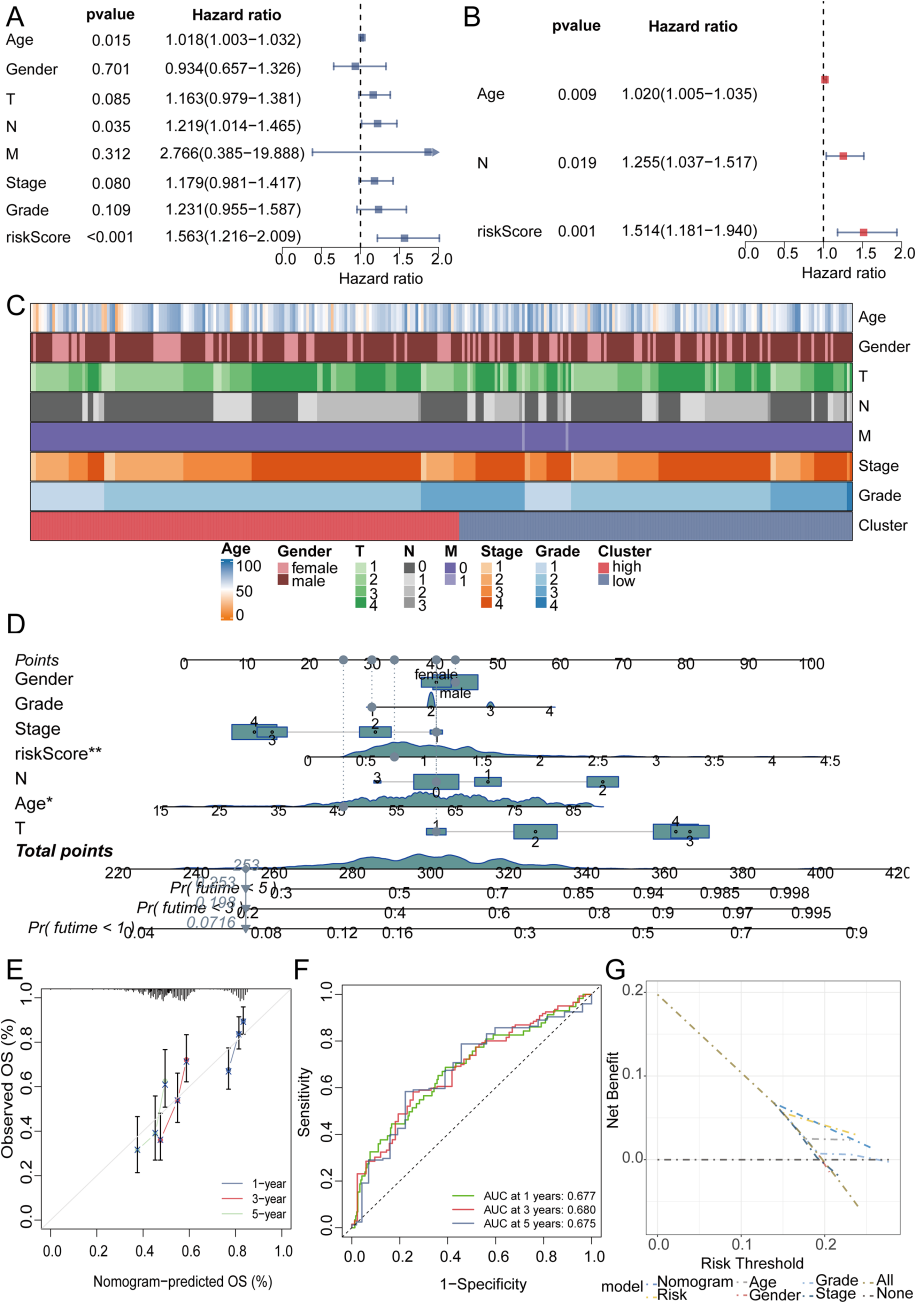
The efficacy validation of the riskScore model and the construction and validation of clinical predictive models. **(A, B)** Univariate and multivariate COX analyses revealed that the riskScore is a valuable independent prognostic factor. **(C)** Heatmap analysis revealed that the high and low risk groups were not related to the patients’ clinical characteristics. **(D)** The nomogram was constructed by integrating the riskScore and clinical factors to predict patient survival at 1, 3, and 5 years. **(E)** The calibration curve illustrates that the model can reasonably predict patient survival. **(F)** The ROC curves displayed the AUC value of the nomogram score. **(G)** DCA curve showed the effectiveness of the clinical prediction model.

We initiated a comprehensive analysis to explore potential interactions among intersecting genes, leveraging the STRING database (**Figure 3A**). Subsequently, A potent prognostic signature was constructed from the 37 candidate genes using Lasso Cox regression. This method enhances model generalizability by automatically selecting predictive features and regularizing coefficients to prevent overfitting. The optimal model, determined by the λ criterion, comprised three genes (MNAT1, PSMD10, and EIF3F), which yielded the best predictive performance (**Figures 3B, C**). To validate the model’s predictive performance, we utilized three datasets. The TCGA dataset was partitioned into training and validation subsets via the “caret” R package, while the GSE41613 oral cancer dataset provided an additional validation set. Consistently across these datasets, patients assigned high-riskScore demonstrated a poorer prognosis. (**Figures 3D–F**). Additionally, survival analysis comparing patient groups stratified by risk revealed a significantly elevated mortality rate in the high-risk cohort. Expression heatmaps further identified markedly higher levels of MNAT1, PSMD10, and EIF3F in the high-risk group relative to the low-risk group (**Figures 3G–I**). To evaluate the link between the riskScore and clinical characteristics, univariate and multivariate Cox regression analyses were performed. Univariate analysis identified age, N stage, and riskScore as significant prognostic indicators. Importantly, multivariate Cox regression confirmed the riskScore as a valuable independent prognostic factor (**Figures 4A, B**). Furthermore, assessment of clinical indicators across the high- and low-risk groups showed no significant differences in these parameters between the cohorts (**Figure 4C**). Recognizing the potential enhancement in prognosis prediction accuracy through the integration of clinical indicators and risk scores, we built a nomogram for 1-, 3-, and 5-year prognosis prediction (**Figure 4D**). Calibration curves demonstrated the nomogram’s favorable predictive performance. ROC curve analysis further validated the effectiveness of the nomogram score, with AUC values at three years of 0.677, 0.680, and 0.675. Moreover, DCA analysis affirmed the nomogram score’s efficacy in predicting prognosis (**Figures 4E–G**).

### 3.3 Immune landscape of RiskScore model

**Figure 5:**
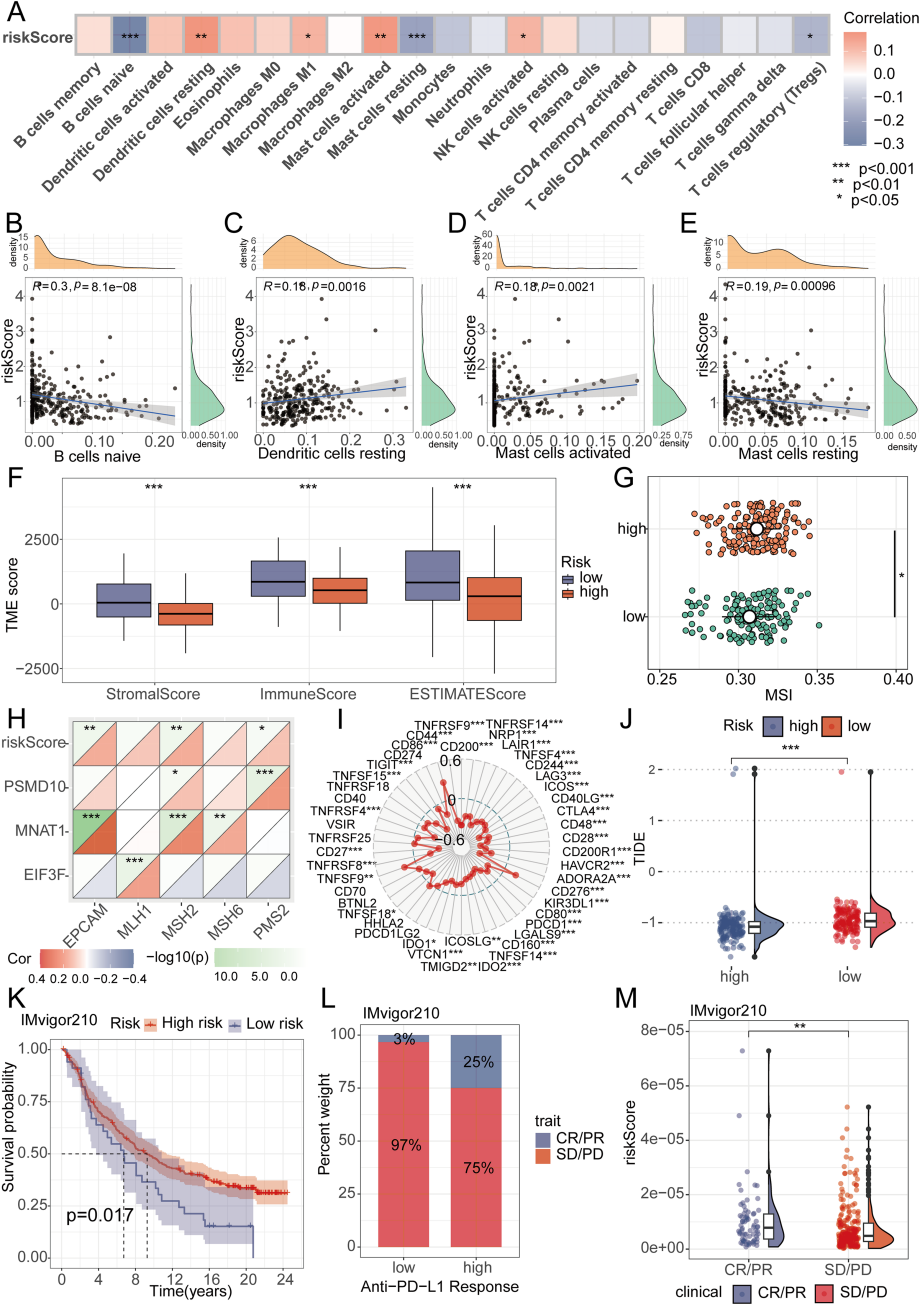
Analysis of RiskScore and immune landscape. **(A)** The correlation between riskScore and 21 immune cells was calculated using the ssGSEA algorithm. **(B-E)** Correlation analysis found that B cells naive, Mast cells resting, Dendritic cells resting, and Mast cells activated had the highest correlation with riskScore (P<0.01). **(F)** Differential analysis found that the high-risk group had lower immune scores, stromal scores, and total scores. **(G)** Differential analysis found that the MSI score was higher in the high-risk group. **(H)** MMR genes were found to be closely associated with riskScore. **(I)** Radar plots showed the correlation between riskScore and multiple immune checkpoints. **(J)** Differential analysis found that the TIDE score was higher in the low-risk group. **(K-M)** Patients with higher riskScore were more likely to experience remission according to the IMvigor210 dataset.

Utilizing the “CIBERSORT” algorithm, we characterized the composition of 21 distinct immune cell populations. Analysis revealed a significant inverse relationship for B cells naive, Mast cells resting, and regulatory T cells (Tregs) with the riskScore; conversely, Dendritic cells resting, Macrophages M1, Mast cells activated, and NK cells activated showed a positive association (**Figure 5A**). To further validate this finding, we performed an in-depth analysis focusing on immune cells displaying P-values below 0.01, demonstrating the association of B cells naive, Mast cells resting, Dendritic cells resting, and Mast cells activated with the riskScore (**Figures 5B–E**). TME as the peripheral environment of tumor cell growth contains various complex components, such as blood vessels, immune cells, fibroblasts, inflammatory cells of bone marrow origin, signaling molecules, and the extracellular matrix (22). Employing the Estimate algorithm, we observed significantly depressed immune, stromal, and ESTIMATE score in the high-risk group compared to the low-risk group (**Figure 5F**). Additionally, further analysis of MSI scores revealed elevated levels in the high-risk group (**Figure 5G**). We utilized MMR and immune checkpoint analysis to predict the potential association between riskScore and immune therapy. EPCAM, MSH2, and PMS2 expression levels showed a positive association with the riskScore based on MMR analysis (**Figure 5H**). Assessment of immune checkpoints further revealed associations between the riskScore and multiple checkpoint indicators. Notably, CTLA4 and PD1 exhibited a close correlation with the riskScore, while PDL1 did not show significant correlation when the p-value threshold was set to 0.001 (**Figure 5I**). We then further evaluated TIDE scores in the high-low risk group, and the results showed that patients in the low-risk group had higher TIDE scores (**Figure 5J**). To assess the therapeutic response among patients stratified by riskScore, we conducted an immune therapy score analysis. The results revealed better treatment outcomes in patients from the high-risk group after receiving immune therapy (**Figures 5K–M**).

### 3.4 Risk model mutation analysis and drug sensitivity analysis

**Figure 6:**
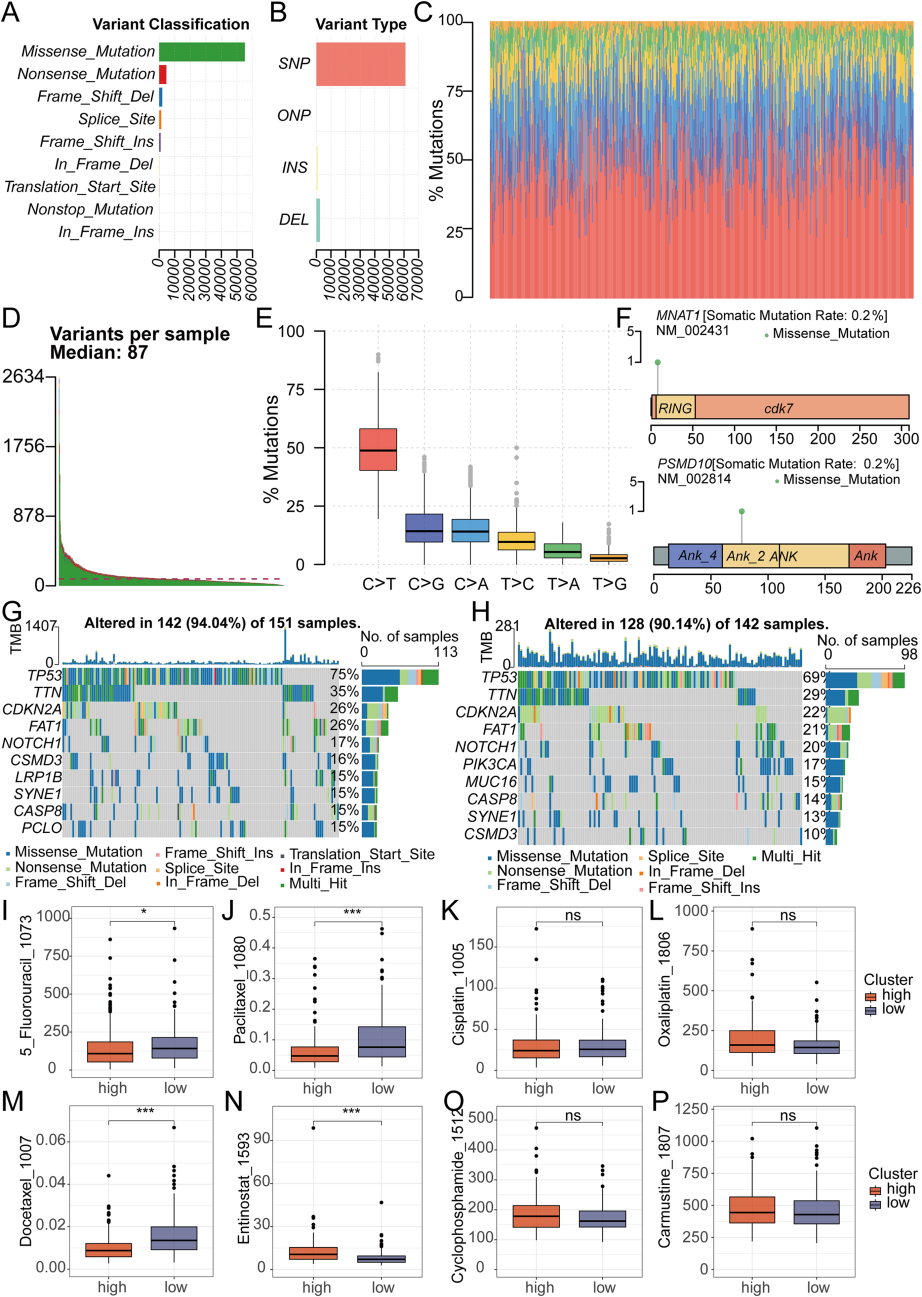
Analysis of Mutation and Drug Sensitivity in RiskScore. **(A-C)** Mutation analysis shows the type of gene mutation in OSCC patient. **(D)** Mutation analysis showed that the median mutation in OSCC patients was 87. **(E)** Mutation analysis showed the type and number of point mutations in OSCC patients. **(F)** Mutation analysis showed the mutation sites of 2 key genes in the model. **(G, H)** The waterfall plot revealed different mutated genes and mutation rates between the high and low-risk groups. **(I-P)** The IC50 of Entinostat was lower in the low-risk group, while it was lower for 5-Fluorouracil, Paclitaxel, and Docetaxel in the high-risk group. There was no significant difference in the IC50 between the two groups for Oxaliplatin, Carmustine, Cyclophosphamide and Cisplatin.

We conducted a thorough analysis of TCGA-derived mutation data for oral cancer patients. Through variant classification analysis, we found that the main mutation category was Missense Mutation (**Figure 6A**). Further variant type analysis revealed single nucleotide polymorphisms as the predominant variant class, revealing an average of 87 mutations per sample (**Figures 6B–D**). For single nucleotide variations, the main type was the C>T transition (**Figure 6E**). To gain deeper insight into the mutational landscape of key model genes, the somatic mutation rate for each gene was separately analyzed. It is worth noting that the mutation rates of MNAT1 and PSMD10 were both 0.2% (**Figure 6F**). Comparative analysis of mutated genes across risk groups revealed significantly elevated mutation frequencies for CSMD3, LRP1B, SYNE1, CASP8, and PCLO in high-risk patients (**Figures 6G, H**). Subsequent assessment of chemotherapy drug sensitivity identified differential responses: The low-risk group demonstrated reduced sensitivity to 5-Fluorouracil, Paclitaxel, and Docetaxel, evidenced by higher IC50 values. Conversely, Entinostat showed diminished efficacy in the high-risk group, reflected in elevated IC50 values (**Figures 6I–P**).

### 3.5 Functional enrichment analysis

In order to deeply understand the differences between high and low-risk groups, we conducted molecular function (MF), biological process (BP), and cellular component (CC) analyses within the Gene Ontology (GO) enrichment framework. In the BP analysis, significantly enriched terms were mainly associated with the biogenesis of ribonucleoprotein complexes, ribosome biogenesis, and cytoplasmic translation (**Supplementary Figure S2A**). MF analysis mainly revealed structural constituent of ribosome, ribonucleoprotein complex binding, and cadherin binding (**Supplementary Figure S2B**). In the CC analysis, significantly enrichments comprised ribosomal subunit, ribosome, and mitochondrial protein-containing complex (**Supplementary Figures S2C, D**). To identify mechanisms underlying prognostic disparities, we conducted KEGG enrichment analysis. The analysis results showed that the main enriched pathways included ribosome, cell cycle, cGMP-PKG signaling pathway, and Rap1 signaling pathway (**Supplementary Figure S2E**). In addition, we also performed hallmark analysis, revealing multiple significantly enriched pathways, including complement system, upregulation of KRAS signaling pathway, PI3K-AKT-MTOR signaling pathway, TGF-β signaling pathway, and TNFα signaling pathway via NFKB (**Supplementary Figure S2F**).

### 3.6 Epithelial cell heterogeneity and pseudo-temporal analysis

**Figure 7:**
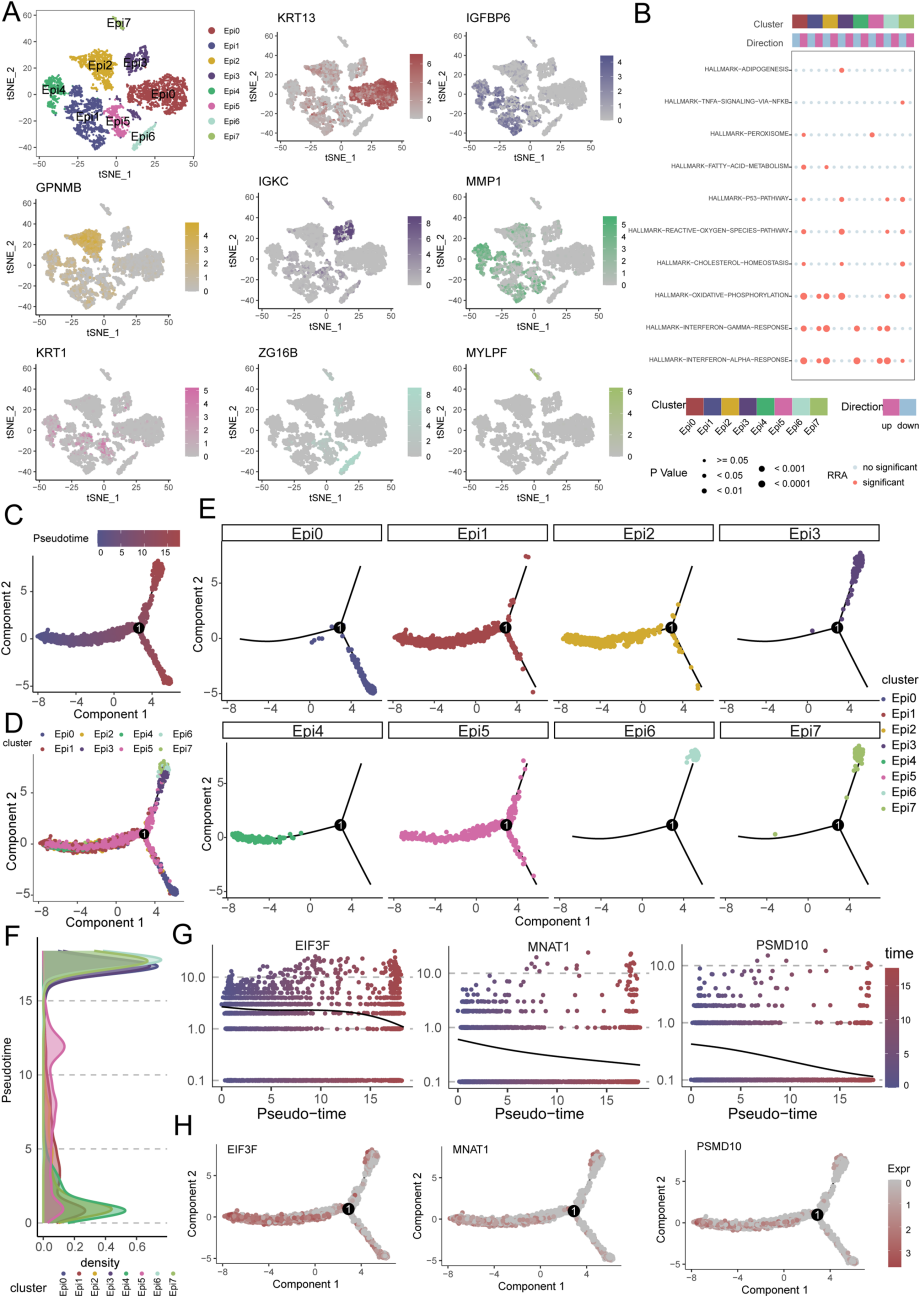
Single-cell transcriptomics reveals epithelial heterogeneity, pathway activation, and pseudotemporal dynamics in OSCC. **(A)** Eight distinct epithelial subpopulations (Epi0-Epi7) were identified from OSCC cellular subpopulations. **(B)** Functional enrichment analysis shows significant activation of oxidative phosphorylation (OXPHOS) and interferon-α/γ response pathways in epithelial subpopulations. **(C, D)** Pseudotime trajectory reconstruction reveals a continuous differentiation continuum across epithelial subpopulations. **(E, F)** Pseudotemporal ordering and density plots position Epi1, Epi2, Epi4 and Epi5 in early pseudotime domains, with Epi0 located in terminal branches. **(G, H)** Expression dynamics of key regulators (EIF3F, MNAT1, PSMD10) along pseudotime, showing high expression in early phases followed by progressive downregulation.

To delineate the functional heterogeneity of epithelial cells in OSCC, we performed single-cell transcriptomic analysis on purified epithelial populations. Unsupervised clustering partitioned these cells into eight distinct clusters(Epi0-Epi7), each exhibiting unique marker gene signatures (**Figure 7A**). Functional enrichment analysis revealed significant activation of oxidative phosphorylation and interferon alpha/gamma response pathways across subsets (**Figure 7B**). Pseudotime trajectory reconstruction uncovered a differentiation continuum spanning these clusters (**Figures 7C, D**). Epi1, Epi2, Epi4, and Epi5 localized to the early pseudotemporal domain, whereas Epi0 occupied terminal positions, suggesting distinct maturation state (**Figures 7E, F**). Notably, critical regulators including EIF3F, MNAT1, and PSMD10 demonstrated stage-specific expression patterns. These genes exhibited peak transcriptional activity in early pseudotime compartments with progressive downregulation along the trajectory, implicating their potential roles in initiating malignant transformation (**Figures 7G, H**).

### 3.7 Functional validation of MNAT1 in OSCC pathogenesis

**Figure 8:**
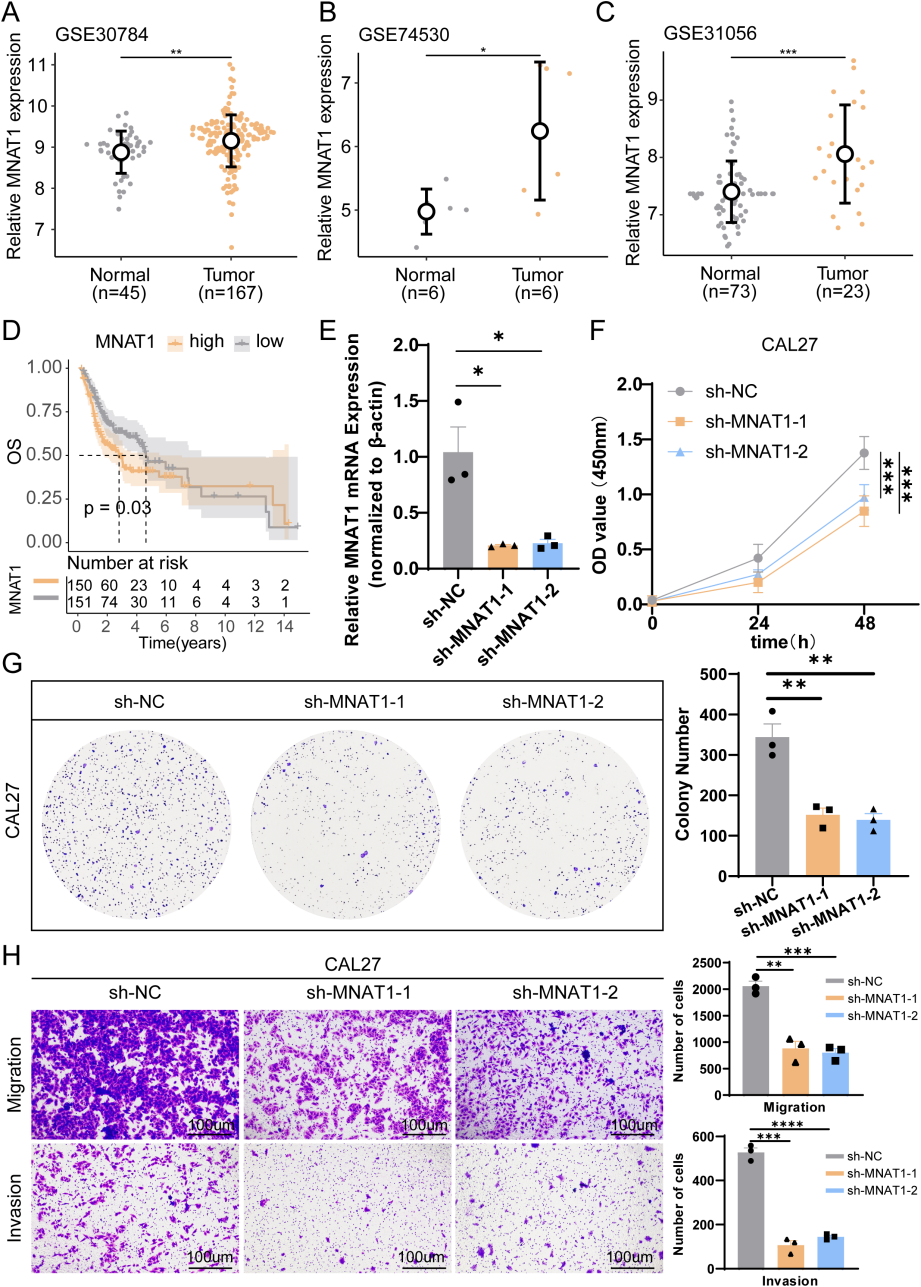
Multi-cohort validation and functional experiment confirm MNAT1 as an oncogenic driver in OSCC. **(A-C)** MNAT1 expression is significantly upregulated in OSCC tumor tissues compared to normal tissues across three independent transcriptomic cohorts. **(D)** Kaplan-Meier analysis demonstrates reduced overall survival in OSCC patients with high MNAT1 expression versus low MNAT1 expression. **(E)** RT-qPCR confirms efficient MNAT1 knockdown in CAL27 cells. n = 3 biologically independent experiments. Data represent mean ± SD. P values were calculated by two-side Student’s t-test. **(F, G)** CCK-8 and colony formation assays reveal significantly impaired proliferative capacity in MNAT1-knockdown CAL27 cells. The quantitative analysis is shown on the right. n = 3 biologically independent experiments. Data represent mean ± SD. P values were calculated by two-side Student’s t-test. **(H)** Transwell migration and Matrigel invasion assays show markedly reduced migratory and invasive abilities following MNAT1 knockdown. Scale bar = 100um. The quantitative analysis is shown on the right. n = 3 biologically independent experiments. Data represent mean ± SD. P values were calculated by two-side Student’s t-test. (*p<0.05; **p<0.01; ***p<0.001; ****p<0.0001).

To substantiate the oncogenic role of MNAT1 in oral carcinogenesis, we first conducted multi-cohort validation across three independent transcriptomic datasets. Consistently, MNAT1 exhibited significant upregulation in tumor tissues compared to normal counterparts (**Figures 8A–C**). Furthermore, elevated MNAT1 expression correlated with poorer patient prognosis (**Figure 8D**). To validate MNAT1’s oncogenic function, we employed the CAL27 OSCC cell line model. Efficient MNAT1 silencing was achieved through shRNA-mediated knockdown (**Figure 8E**). Subsequent CCK-8 proliferation assays revealed a time-dependent attenuation of cell viability, with optical density (OD450) decreasing at 48 hours post-transfection compared to scramble controls (**Figure 8F**). This anti-proliferative effect was also corroborated by colony formation assays (**Figure 8G**). Furthermore, Transwell migration and invasion assays exhibited significant decreases respectively upon MNAT1 suppression (**Figure 8H**).

### 3.8 Macrophage-epithelial crosstalk in OSCC progression

**Figure 9:**
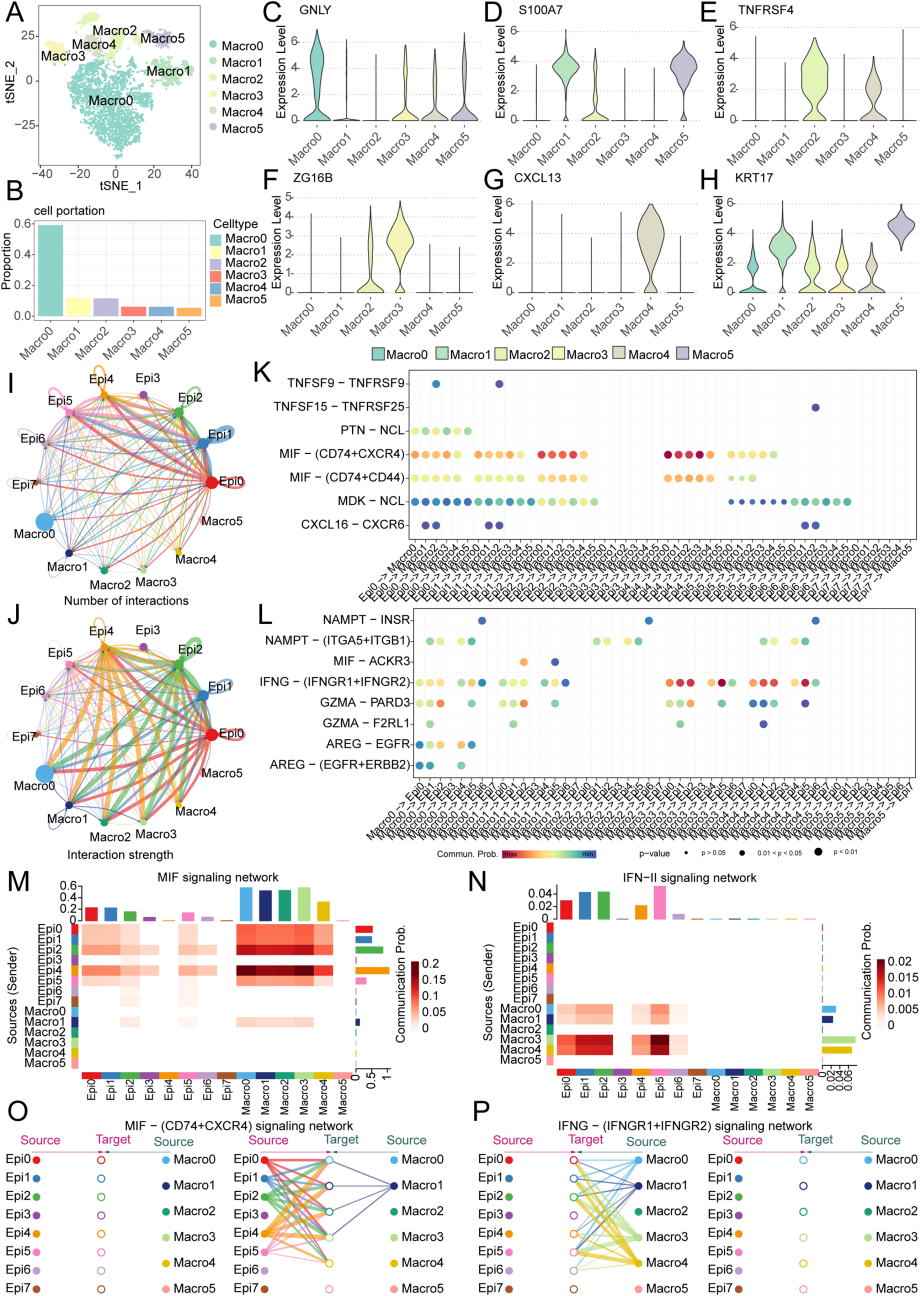
Single-cell analysis reveals macrophage heterogeneity and epithelial-macrophage crosstalk driving OSCC progression. **(A)** Five distinct macrophage subpopulations (Macro0-Macro4) were identified in OSCC. **(B)** Bar plot demonstrates the proportional distribution of macrophage subsets, with Macro0 representing the predominant population. **(C-H)** Violin plots display canonical marker gene expression profiles for each macrophage subpopulation. **(I, J)** Cell communication analysis quantifies interaction number and strength between epithelial and macrophage subsets. **(K, L)** Dot plots visualize significant ligand-receptor pairs across epithelial and macrophage subpopulations. **(M, N)** Dominant directional signaling axes. **(M)** MIF pathway mediates epithelial-to-macrophage signaling; **(N)** IFN-II (IFNγ) pathway drives macrophage-to-epithelial communication. **(O, P)** Key ligand-receptor interactions: **(O)** MIF-(CD74+CXCR4) primarily links Epi0/Epi1/Epi2/Epi4/Epi5 to Macro0/Macro1/Macro2/Macro3/Macro4; **(P)** IFNγ-(IFNGR1+IFNGR2) mainly connects Macro0/Macro1/Macro3/Macro4 with Epi0/Epi1/Epi2/Epi4/Epi5/Epi6.

To further investigate the functional interplay between tumor cells and macrophages, we moved beyond the conventional M1/M2 classification paradigm. Using unsupervised single-cell clustering, we resolved macrophage heterogeneity into six transcriptionally distinct subsets (Macro0-Macro5), with Macro0 representing the predominant population (**Figures 9A, B**). Subtype-specific marker gene signatures were systematically annotated (**Figures 9C–H**). CellChat analysis revealed intensive bidirectional communication between epithelial and macrophage subsets, with quantitative mapping of interaction number and strength network (**Figures 9I, J**).

Intercellular communication networks were revealed through ligand-receptor pair analysis, revealing bidirectional signaling between epithelial and macrophage subsets (**Figures 9K, L**). It can be seen that the main pathway from epithelial cells to macrophages is the MIF signaling network, while the main pathway from macrophages to epithelial cells is the IFN-II signaling pathway (**Figures 9M, N**, **Supplementary Figures S3A, B**). Moreover, the main ligand-receptor pairs in the MIF pathway are the MIF - (CD74+CXCR4) signaling network, while the main ligand-receptor pairs in the IFN-II pathway are the IFN-(IFNGR1+IFNGR2) signaling pathway (**Supplementary Figures S3C, D**). Specifically, in the MIF - (CD74+CXCR4) signaling network, it can be seen that the interactions mainly occur between Epi0, Epi1, Epi2, Epi4, Epi5 and Macro0, Macro1, Macro2, Macro3, whereas in the IFN-(IFNGR1+IFNGR2) signaling pathway, the interactions primarily involve Macro0, Macro1, Macro3, Macro4 and Epi0, Epi1, Epi2, Epi4, Epi5, Epi6 (**Figures 9O, P**, **Supplementary Figures S3E–H**). We made an interesting observation through cell-cell communication analysis: compared to other clusters of tumor epithelial cells, clusters EPI12, EPI4, and EPI5 prominently interact with macrophages via the MIF signaling pathway. Notably, MNAT1 is highly expressed precisely in these subpopulations, suggesting that MNAT1 may facilitate crosstalk with macrophages through the MIF pathway. To test whether MNAT1 functionally influences this interaction, we knocked down MNAT1 and observed a significant decrease in MIF expression (**Supplementary Figure S4A**). We next explored the functional consequence of this regulation using a co-culture system. THP-1 cells were first differentiated into M0 macrophages by PMA treatment for 24 hours, and then co-cultured with tumor cells. Following co-culture, macrophages were collected and analyzed. Strikingly, macrophages co-cultured with MNAT1-knockdown tumor cells showed reduced expression of the M2 markers CD206 and CD163 (**Supplementary Figures S4B, C**), indicating that MNAT1 knockdown suppresses M2 polarization of macrophages, thereby impeding tumor progression.

## 4 Discussion

OSCC, as a complex and variable disease, faces many challenges in the fields of treatment and research (23). Although traditional surgical, radiotherapy, and chemotherapy still dominate, with the rapid development of medical technology, targeted therapy and immunotherapy are gradually integrating into clinical practice, bringing new treatment options for patients (24). However, the diversity and heterogeneity of OSCC lead to significant differences in the efficacy of targeted therapy and immunotherapy among different patients (4). Therefore, we urgently need more accurate methods to predict patient prognosis and formulate personalized treatment plans (25). To this end, we have conducted in-depth studies on the role of hub genes in OSCC and constructed a prognosis model based on these genes. By analyzing patients’ gene expression data, we can more accurately predict patient prognosis, provide more accurate survival predictions and personalized treatment plans for clinical practice, thereby potentially improving patient survival rates and quality of life.

In this study, given the close association between T cells and oral cancer, we used single-cell sequencing analysis to screen T cell marker genes. Additionally, considering the important role of ubiquitination genes in oral cancer patients, we also included ubiquitination modification genes, applying WGCNA integrated with differential and prognostic approaches. This integrative strategy enabled construction of a predictive model featuring key genes such as MNAT1, PSMD10, and EIF3F. While the expression patterns, functional significance, and underlying mechanisms of MNAT1 remain entirely unexplored in OSCC, it has been demonstrated to promote proliferation and cisplatin resistance in osteosarcoma by modulating the PI3K/Akt/mTOR pathway (26). Furthermore, PSMD10 is regarded as a biomarker for epithelial carcinogenesis, and overexpression has been observed in human oral cancer. And, consequently, HNSCC patients with relatively high PSMD10 expression levels have a shorter survival period (27, 28). Although the relationship between EIF3F and OSCC is similarly uninvestigated, its tumor-promotional functions are emerging in other malignancies, where it has been shown to enhance migration and invasion in lung cancer and to remodel fatty acid biosynthesis to fuel malignancy in hepatocellular carcinoma (29, 30). These studies have confirmed from multiple perspectives the close relationship between these genes and cancer, further validating the reliability of the selected genes in the predictive model we established. The riskScore model demonstrated favorable prognostic performance, offering valuable clinical utility for treatment planning and patient management.

Given the significant impact of immune cells in the tumor microenvironment of oral cancer, we conducted an in-depth analysis of 21 immune cell subpopulations. Among them, the subpopulations of B cells naive, Mast cells resting, Dendritic cells resting, and Mast cells activated particularly attracted our attention. Extensive research has confirmed that B cells, dendritic cells, and mast cells are closely related to the development of OSCC (31–33). Moreover, MSI, MMR, and immune checkpoints have become significant predictors for immunotherapy (34, 35). In our study, the high-risk group showed higher MSI scores. This finding piqued our interest, as patients with high MSI expression often demonstrate better treatment outcomes in immunotherapy (36). It suggests that high-risk patients might have a poorer prognosis without immunotherapy but could respond more positively to immunotherapy. Further MMR correlation analysis revealed a strong correlation between the riskScore and genes such as EPCAM, MSH2, and PMS2, which further supports the potential value of our riskScore in predicting patient responses to immunotherapy. To validate this, we conducted an immunotherapy response analysis, and the results indicated that patients responsive to immunotherapy had higher riskScore, consistent with our previous analysis. Therefore, we found that OSCC patients with poorer prognosis might benefit more from immunotherapy. These findings provide new perspectives and evidence for personalized treatment strategies for oral cancer.

Given the established contribution of gene mutations to oral cancer pathogenesis, comprehensive mutation profiling revealed significantly elevated mutational frequencies for TP53, TTN, FAT1, CSMD3, LRP1B, and PCLO in high-risk relative to low-risk patients. Among these, mutations in the P53 gene have been shown to regulate the immune microenvironment in OSCC (37), underscoring its critical influence on the progression of oral cancer. Additionally, mutations in the TTN gene were identified in metastatic OSCC patients, suggesting its involvement in the metastasis process (38). The FAT1 gene suppresses carcinogenesis, modulates oxidative stress, and potentiates cisplatin response in OSCC via the LRP5/WNT2/GSS signaling cascade (39). Similarly, mutations in CSMD3, LRP1B, and PCLO are closely associated with tumor development and progression (40–42). These findings deliver compelling insights into genetic distinctions across risk-stratified cohorts, enhancing our understanding of oral cancer pathogenesis while informing future precision therapeutics.

Chemotherapy, a cornerstone in cancer treatment, has seen significant advancements over the past few decades (43). To assess differential chemotherapy responses across risk-stratified cohorts, we performed a detailed analysis of eight common chemotherapeutic agents used in oral cancer treatment. The results indicated that the IC50 values for 5-Fluorouracil, Paclitaxel, and Docetaxel were relatively higher in the low-risk group. Conversely, in the high-risk group, the IC50 value for Entinostat was notably higher. By systematically analyzing the IC50 values of different chemotherapy drugs across the two patient groups, we provided robust scientific evidence and guidance for selecting the most appropriate chemotherapeutic agents for distinct patient cohorts in future clinical practice.

To uncover molecular mechanisms driving prognostic disparities in risk-stratified cohorts, we performed comprehensive functional enrichment profiling. At the BP level, we observed significant enrichment in cytoplasmic translation and rRNA processing. At the CC level, ribosome and cell-substrate junction were notably enriched, while at the MF level, cadherin binding, translation factor activity, and RNA binding were prominently highlighted. Current research substantiates the crucial roles of ribosomes, rRNA processing, and cadherin binding in tumorigenesis and cancer progression (44–46). Additionally, the enrichment of cell-substrate junction and cadherin binding functions suggests their potential involvement in tumor metastasis (47, 48). KEGG pathway analysis revealed significant enrichment in the Ribosome and Cell Cycle pathways, both critically involved in OSCC pathogenesis (49, 50). Furthermore, HALLMARK enrichment profiling demonstrated significant enrichments in the TGF-β transduction pathway, PI3K/AKT/mTOR signaling cascade, and TNFα-NFκB signaling axis. Literature indicates that OSCC cells interact with cancer-associated fibroblasts (CAFs) through the TGF-β/SOX9 axis during cancer progression (51). Our study also found that tumor-promoting CAFs with high itgb2 expression can activate the PI3K/AKT/mTOR axis, thereby promoting OSCC tumor proliferation via NADH-driven oxidative phosphorylation in mitochondria (52). Additionally, TNF-α and lipopolysaccharide (LPS) play critical roles in inflammation regulation during tumorigenesis (53).These findings not only reinforce our analysis results, providing strong evidence for prognostic disparities across risk-stratified cohorts but also offer valuable insights into potential mechanistic pathways, guiding future research in exploring therapeutic targets.

Subsequently, to further clarify the role of key genes in tumor epithelial cells, we performed pseudotime analysis. Typically, pseudotime analysis transitions from a low differentiation state to a high differentiation state. Our results suggest that MNAT1, EIF3F, and PSMD10 are all highly expressed in the low differentiation state, indicating these key genes are critical factors promoting tumor malignancy. Through literature review, we found that MNAT1 plays an important role in tumor development. Therefore, we focused on MNAT1. Through CCK8, colony formation, and Transwell experiments, we further confirmed that MNAT1 promotes OSCC proliferation and migration. Further cell communication analysis suggests that tumor epithelial cells and macrophages primarily interact through the MIF and IFN-II pathways. Studies have shown that the importance of MIF and IFN-II in cancer has been confirmed in many clinically relevant cancer models and is closely related to cancer development (54, 55). These studies further support our findings, indicating that MNAT1 in tumor epithelial cells promotes M2 macrophage polarization via the MIF-(CD74+CXCR4) pathway, thereby driving OSCC progression.

Certainly, our study has several limitations. First, although we utilized multiple public databases to construct and validate the prediction model, some datasets may lack comprehensive clinical details. To more accurately elucidate the nature of the disease and its predictors, we plan to prospectively collect samples and data from our hospital in future work, thereby filling this gap through in-depth analysis. Second, while the model demonstrates excellent performance within the current study cohort, its clinical applicability requires further consideration. Specifically, our cohort was derived from a single institution with a relatively homogeneous ethnic background. Significant differences in genetics, lifestyle, and environmental factors across different ethnicities and regions may influence gene expression patterns and, consequently, the performance of our gene signature. We aim to further validate the model using local patient cohorts in subsequent studies. Finally, our model is mainly based on patients who have received initial treatment, rather than those who have undergone multiple rounds of chemotherapy and immunotherapy. This may impose limitations on the assessment of patients. In the future, we hope to further collect data from patients with various treatment backgrounds to build a more accurate model.

Overall, this study has important clinical application value. The prognostic model constructed by multiple ubiquitination modification and T cell-related genes can more accurately predict the survival of patients, and this model can further predict the efficacy of chemotherapy and immunotherapy, providing guidance for the selection of clinical drugs. Through single-cell sequencing, we identified MNAT1 as a master oncogenic driver that potentiates OSCC proliferation and metastasis. Furthermore, we found that MNAT1 directly influences macrophage polarization through MIF-mediated epithelial-macrophage crosstalk, thereby impacting OSCC progression.

## 5 Conclusion

We present a ubiquitination and T cell-based prognostic model predicting OSCC survival and treatment response. We have further validated MNAT1 as an oncogenic driver and defined its role in promoting macrophage M2 polarization through MIF-mediated epithelial-macrophage crosstalk.

## Data Availability

The original contributions presented in the study are included in the article/**Supplementary Material**. Further inquiries can be directed to the corresponding authors.

## Supplementary material

Supplementary Figure 1 Single-cell sequencing analysis for screening T cell-related genes. **(A-C)** The violin plots show the nFeature_RNA, nCount_RNA, and percent_MT of 6 OSCC samples. **(D)** The scatter plot shows that the correlation between nFeature_RNAh and nCount_RNA is 0.89. **(E)** The volcano plot displays 1500 highly variable genes. **(F)** The heatmap displays the major differentially expressed genes in different cell types.

Supplementary Figure 2 GO, KEGG, and HALLMARK functional enrichment **(A-D)** Gene Ontology enrichment analysis of the riskScore model. **(E)** KEGG enrichment analysis of the riskScore model. **(F)** HALLMARK enrichment analysis of the riskScore model.

Supplementary Figure 3 Single-cell analysis reveals epithelial-macrophage communication networks in OSCC. **(A, B)** Heatmaps quantify interaction weights of senders, receivers, mediators, and influencers within the MIF and IFN-II signaling pathways. **(C, D)** Violin plots visualize dominant ligand-receptor pairs mediating cellular crosstalk in MIF and IFN-II pathways. **(E-H)** Circos diagrams map primary cellular interactions for: **(E)** MIF-ACKR3 signaling; **(F)** MIF-(CD74+CXCR4) signaling; **(G)** MIF-(CD74+CD44) signaling; **(H)** IFNγ-(IFNGR1+IFNGR2) signaling.

Supplementary Figure 4 MNAT1 influenced M2 macrophage polarization. **(A-C)** The expression of MIF, CD206 and CD163 mRNA in TAMs co-cultured with control or MNAT1-KD CAL27 cells, detected by RT-qPCR analyses (n=3). Data represent mean ± SD. P values were calculated by two-side Student’s t-test. (*p<0.05; **p<0.01; ***p<0.001; ****p<0.0001).
