# Supplementary figures and images for: Integrating scRNA-seq and machine learning identifies MNAT1 as a therapeutic target in OSCC
Source: Front Immunol. 2025 Oct 29;16:1663487. doi: 10.3389/fimmu.2025.1663487 (PMC12605385; doi:10.3389/fimmu.2025.1663487)

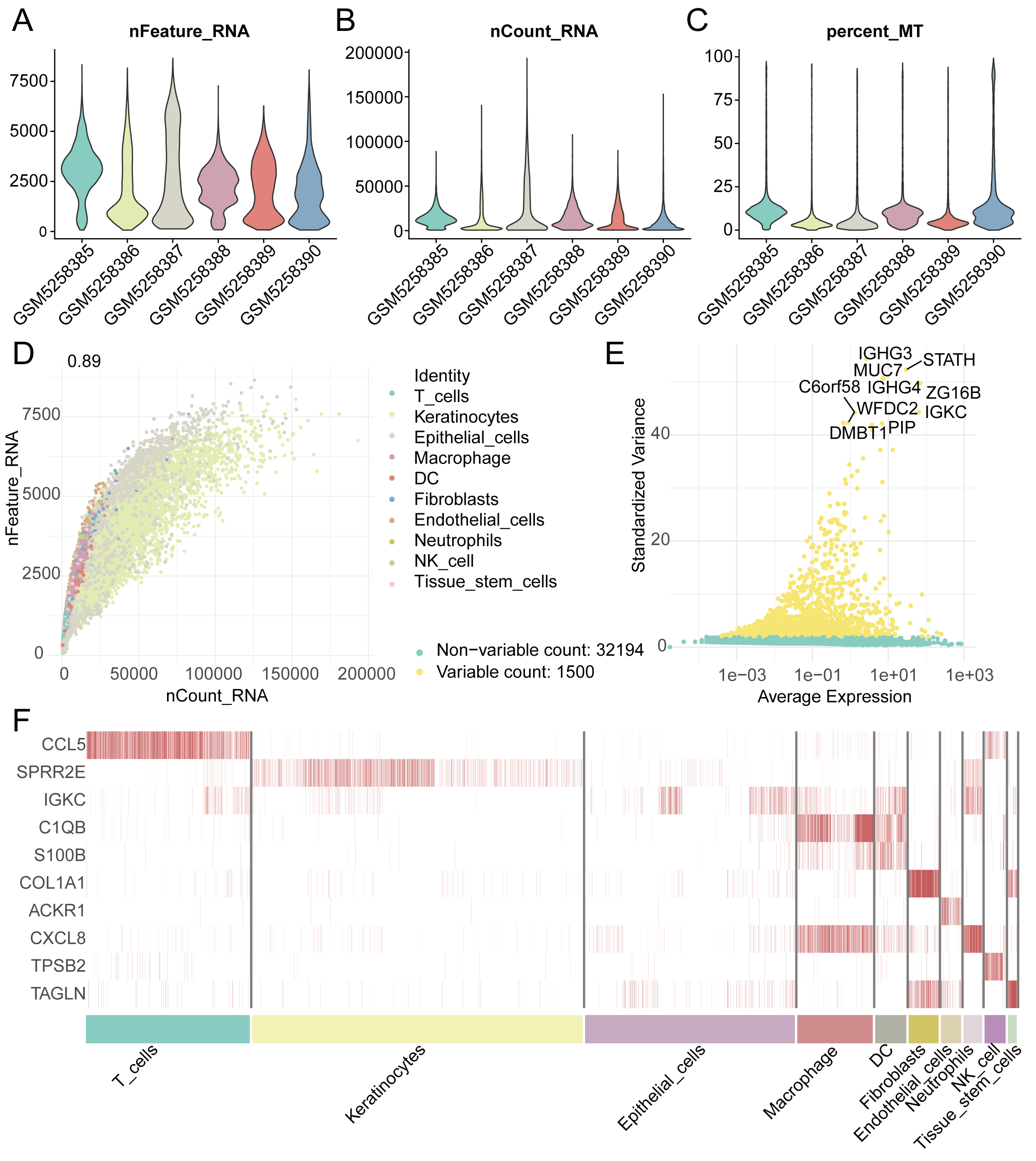

Supplement: Supplementary Figure 1 — Single-cell sequencing analysis for screening T cell-related genes. (A-C) The violin plots show the nFeature_RNA, nCount_RNA, and percent_MT of 6 OSCC samples. (D) The scatter plot shows that the correlation between nFeature_RNAh and nCount_RNA is 0.89. (E) The volcano plot displays 1500 highly variable genes. (F) The heatmap displays the major differentially expressed genes in different cell types. [file Image1.tif]

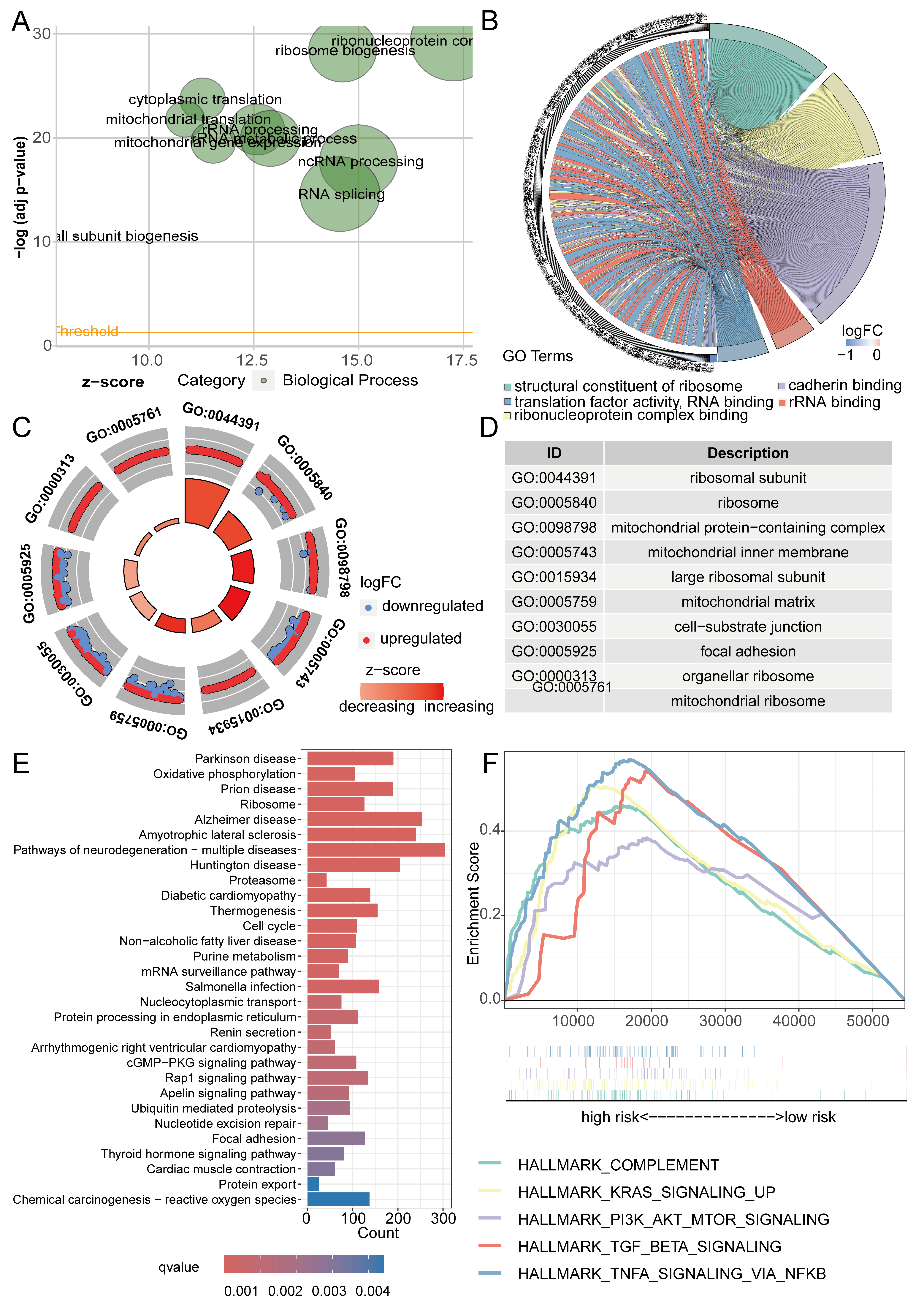

Supplement: Supplementary Figure 2 — GO, KEGG, and HALLMARK functional enrichment (A-D) Gene Ontology enrichment analysis of the riskScore model. (E) KEGG enrichment analysis of the riskScore model. (F) HALLMARK enrichment analysis of the riskScore model. [file Image2.tif]

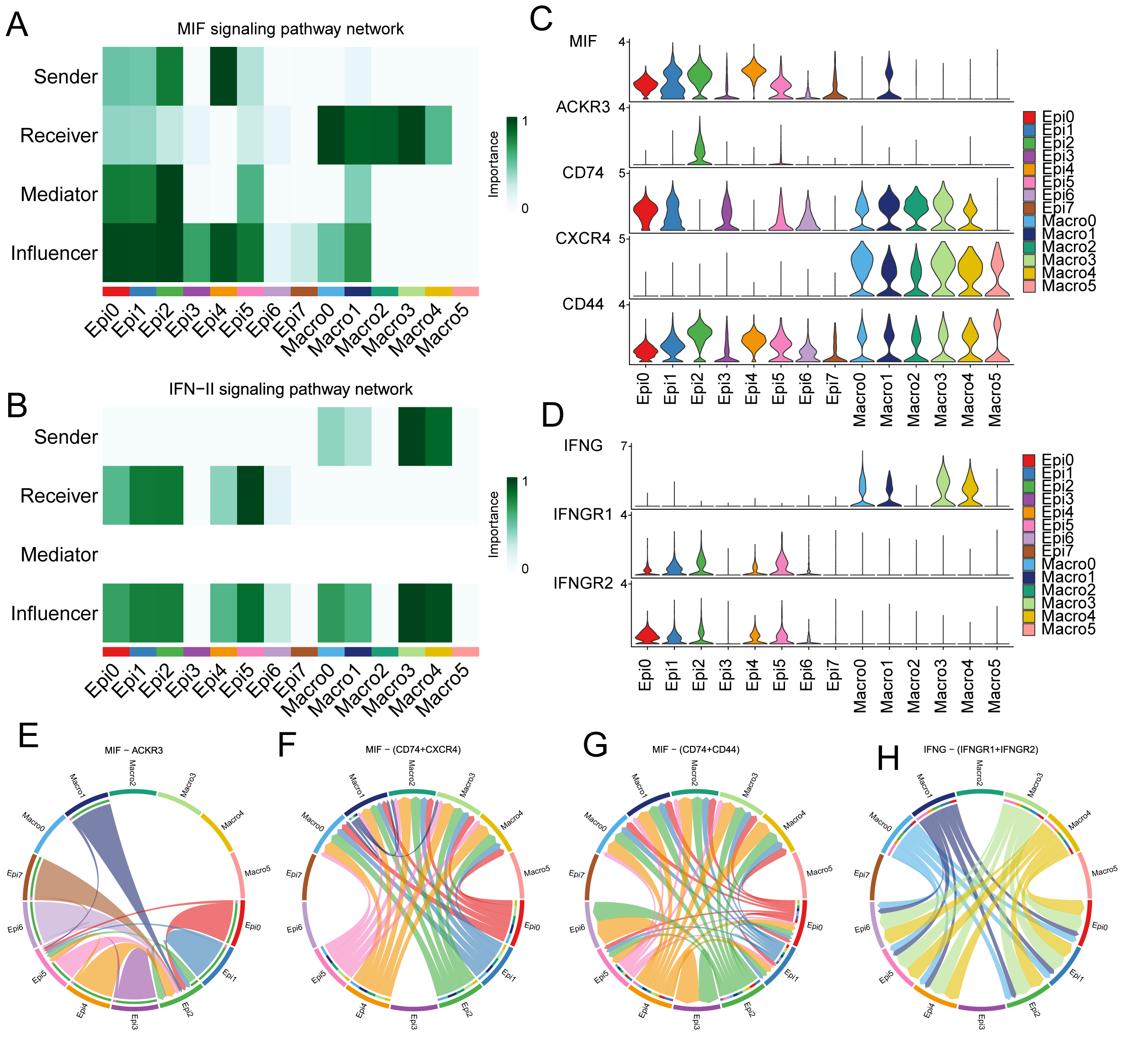

Supplement: Supplementary Figure 3 — Single-cell analysis reveals epithelial-macrophage communication networks in OSCC. (A, B) Heatmaps quantify interaction weights of senders, receivers, mediators, and influencers within the MIF and IFN-II signaling pathways. (C, D) Violin plots visualize dominant ligand-receptor pairs mediating cellular crosstalk in MIF and IFN-II pathways. (E-H) Circos diagrams map primary cellular interactions for: (E) MIF-ACKR3 signaling; (F) MIF-(CD74+CXCR4) signaling; (G) MIF-(CD74+CD44) signaling; (H) IFNγ-(IFNGR1+IFNGR2) signaling. [file Image3.tif]

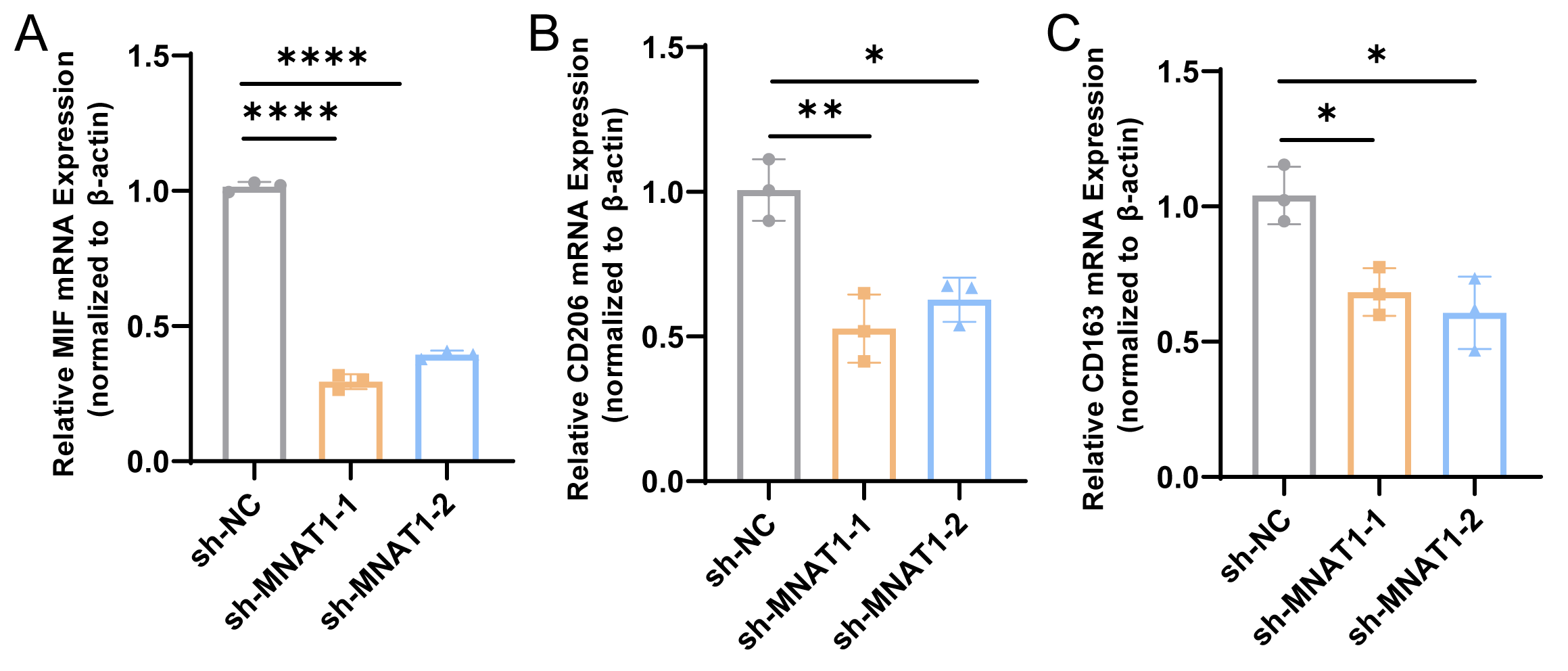

Supplement: Supplementary Figure 4 — MNAT1 influenced M2 macrophage polarization. (A-C) The expression of MIF, CD206 and CD163 mRNA in TAMs co-cultured with control or MNAT1-KD CAL27 cells, detected by RT-qPCR analyses (n=3). Data represent mean ± SD. P values were calculated by two-side Student’s t-test. (*p<0.05; **p<0.01; ***p<0.001; ****p<0.0001). [file Image4.tif]
