# Supplementary material for: Integrating scRNA-seq and machine learning identifies MNAT1 as a therapeutic target in OSCC
Source: Front Immunol. 2025 Oct 29;16:1663487. doi: 10.3389/fimmu.2025.1663487 (PMC12605385; doi:10.3389/fimmu.2025.1663487)
Supplement: Supplementary file 5 [file Table1.docx]

#### Table 1. The sequences of primers used for RT-qPCR assay.

| **Gene name** | **Primer orientation** | **Sequences** |
| --- | --- | --- |
| MNAT1 | Forward | 5′-GGTTGCCCTCGGTGTAAGAC-3′ |
|  | Reverse | 5′-AGTTGCTCTTTCTGAGTGGAGT-3′ |
| GAPDH | Forward | 5′-GAGAAGGCTGGGGCTCATTT-3′ |
|  | Reverse | 5′-AGTGATGGCATGGACTGTGG-3′ |

#### Table 2. The sequences of shRNA.

| **Gene name** | **Sequences** |
| --- | --- |
| shRNA#1 | 5′-CCTAGTCTAAGAGAATACAAT-3′ |
| shRNA#2 | 5′-GCTATACTTCTTCTCTTGCTT-3′ |
